# High expression of high mobility group box 1 (hmgb1) predicts poor prognosis for hepatocellular carcinoma after curative hepatectomy

**DOI:** 10.1186/1479-5876-10-135

**Published:** 2012-07-02

**Authors:** Furong Liu, Yaojun Zhang, Zhenwei Peng, Hengjun Gao, Li Xu, Minshan Chen

**Affiliations:** 1 Department of Hepatobiliary Surgery, Cancer Centre of Sun Yat-Sen University, 651 Dongfeng Road East, Guangzhou, 510060, China; 2 State Key Laboratory of Oncology in Southern China, Guangzhou, 510060, China

**Keywords:** High mobility group box 1, Hepatocellular carcinoma, Hepatectomy, Prognosis, Prognostic factor

## Abstract

**Background:**

High mobility group box 1(HMGB1) overexpression has been reported in a variety of human cancers. However, the role of HMGB1 in hepatocellular carcinoma (HCC) remains unclear. The aim of present study was to analyze HMGB1 protein expression in tumor, para-tumor and normal tissue and to assess its prognostic significance for HCC after curative hepatectomy.

**Methods:**

The levels of HMGB1 mRNA and protein in tumor, para-tumor and normal tissue were evaluated in 11 HCC cases by Reverse Transcription-polymerase chain reaction (RT-PCR) and Western blot. Additionally, HMGB1 protein expression in 161 HCC was analyzed by immunohistochemistry and correlated with clinicopathological characteristics and survivals. Student’s t-test, spearman’s rank correlation, Kaplan-Meier plots and Cox proportional hazards regression model were used to analyze the data.

**Results:**

By RT-PCR and Western blot, the levels of HMGB1 mRNA and protein were significantly higher in HCC, compared to that in para-tumor (*p* < 0.001) and normal tissue (*p* < 0.001). Immunohistochemical staining revealed that high expression of HMGB1 was detected in 42.9% (69/161) HCC cases. High expression of HMGB1 was significantly associated with incomplete encapsulation (*p* = 0.035) and advanced TNM stage (*p* = 0.036). Multivariate analysis showed that high expression of HMGB1 was an independent prognostic factor for both overall (*p* = 0.009, HR = 1.834, 95%CI: 1.167-2.881) and disease-free survival (*p* = 0.018, HR = 1.622, 95%CI: 1.088-2.419), along with tumor size. Subgroup analysis revealed that high expression of HMGB1 predicted poorer overall survival only for tumor >5 cm (*p* = 0.031), but not for tumor ≤5 cm (*p* = 0.101).

**Conclusions:**

HMGB1 protein might contribute to the malignant progression of HCC, high expression of HMGB1 predicts poor prognosis for patients with HCC after curative hepatectomy, especially for patients with tumor >5 cm.

## Background

Hepatocellular carcinoma (HCC) is the fifth most common cancer worldwide and the third most frequent cause of death of cancer. Although the majority of cases are still found in Asia and Africa, recent evidence has shown that the incidence and mortality rate of HCC are rising in North America and Europe [1]. Liver resection is still considered to be the mainly curative therapy for HCC, with about 50-70% 5-year overall survival after curative hepatectomy. However, the postoperative recurrence rate remains as high as 70%-83.7% [2,3]. Therefore, it is critical to identify prognostic factors for patients with HCC after hepatectomy. Unfortunately, at present, we are unable to prognosticate accurately on the basis of commonly used clinicopathological characteristics [1-4].

High mobility group box 1(HMGB1), an evolutionarily ancient protein, is a nuclear DNA-binding protein that loosely binds to chromatin and presents in almost all eukaryotic cells [5]. The nuclear role of HMGB1 is not only to integrate and stabilize nucleosome by means of making DNA bending and facilitating the bind of several regulatory protein complexes to DNA, such as the nuclear factor-κB (NF-κB), p53, p73 transcriptional complexes [6,7], but also to facilitate the integration of transposons [8], to regulate transcriptional activation [9]. Moreover, HMGB1 can be released from necrotic cells, activated macrophages, mature dendritic cells and natural killer cells to mediate late systemic inflammation which makes it one of the main prototypes of the emerging damage-associated molecular pattern molecules [10-12]. HMGB1 plays corresponding roles in cells through its receptors: RAGE (receptor for advanced glycation end-products) and TLRs (Toll like receptors). Recent studies have revealed that the binds of HMGB1 and RAGE or TLRs are involved in the activation of several pathways, such as NF-κB pathway, PI3K/AKT pathway, as well as signal transduction through AKT, ERK and p38 [13-16].

The connection that widely exists between HMGB1 and intracellular signal pathways makes HMGB1 can function during inflammation, cell differentiation and migration, tumor invasion and metastasis [17]. High expression of HMGB1 has been showed to be a strong predictor of poor survivals in kinds of malignancies, including colorectal cancer, gastric cancer, nasopharyngeal carcinoma, and squamous-cell carcinoma of the head and neck et al [18-21]. More recently, Jiang W et al [22] reported that HMGB1 was associated with clinicopathologic features in patients with hepatocellular carcinoma, but the role of HMGB1 in predicting prognosis of HCC after curative hepatectomy remains unclear. In present study, we evaluated the expression of HMGB1 in tumor, para-tumor and normal tissue, to assess its prognostic significance in HCC patients after curative hepatectomy.

## Methods

### Patients and clinical specimens

To detect the mRNA and protein level of HMGB1 in tumor, para-tumor (defined as ≤2.0 cm distance from tumor edge) and normal (defined as >2.0 cm distance from tumor edge) tissue, fresh tissues were collected from 11 patients with HCC who underwent hepatectomy between Sep 2011 and Oct 2011 in our department, Department of Hepatobiliary Surgery, Cancer Centre of Sun Yat-Sen University (Guangzhou, China).

A cohort of consecutive 161 previously untreated patients who received curative hepatectomy for HCC in our department from Jan 2004 to Dec 2005 was enrolled. All patients were confirmed by histological diagnosis. In present study, curative hepatectomy was defined as followed: 1) microscopically complete removal of the tumor; 2) ≤3 tumors, no vascular and bile duct invasion; 3) no lymph node or distance metastasis. For the use of these clinical materials for research purposes, prior patient’s consent and approval from the Ethics Committee of Cancer Centre of Sun Yat-Sen University were obtained.

The main clinical and pathological variables of all patients were described in detail in Table 1. In brief, there were 140 male and 21 female patients, with a median age of 48 years old (mean ± SD: 46.1 ± 11.5, range: 14-70). Tumor size ranged from 1.5 cm to 24.0 cm (mean ± SD: 6.3 ± 4.1), 85 patients (52.8%) had tumor ≤5.0 cm and 76 (47.2%) had tumor >5.0 cm. 147 patients (91.3%) had single tumor and 14 (8.7%) had 2-3 tumors. 144 patients (89.4%) had HBV infection, and only one patient had HCV infection. According to the 7th edition tumor-node-metastasis (TNM) classification of the American Joint Committee on Cancer (AJCC) [23], 131 patients (81.4%) had stage I disease, 11 (6.8%) had stage II disease, and 19 (11.8%) had stage III disease respectively (Table 1).

**Table 1:** Correlations between HMGB1 expression and clinicopathalogic characteristics in 161 patients with HCC

**Variables**	**All Patients (n = 161)**	**High HMGB1 (n = 69)**	**Low HMGB1 (n = 92)**	***P*value**
Age(mean ± SD, year)	46.1 ± 11.5	44.9 ± 11.6	48.1 ± 11.3	0.511
≤55	128	59	69	0.103
>55	33	10	23	
Gender				0.639
Male	140	61	79	
Female	21	8	13	
HBV infection				0.377
Absent	17	9	8	
Present	144	60	84	
AFP level				0.785
≤400 ng/ml	89	39	50	
>400 ng/ml	72	30	42	
Liver cirrhosis				
Absent	22	9	13	
Present	139	60	79	
Child-pugh				0.403
A	158	67	91	
B	3	2	1	
Tumor size(mean ± SD, cm)	6.3 ± 4.1	6.0 ± 3.6	6.5 ± 4.5	0.075
≤5 cm	85	36	49	0.892
>5 cm	76	33	43	
Tumor number				
Single	147	62	85	0.575
2-3	14	7	7	
Tumor encapsulation				0.035
Complete	71	37	34	
Incomplete	90	32	58	
TNM stage				0.036
I	131	51	80	
II-III	30	18	12	
Tumor differentiation				
I-II	67	23	44	0.066
III-IV	94	46	48	

HBV: hepatitis B virus, AFP: alpha fetoprotein, HMGB1: high mobility group box 1.

### Reverse transcription-polymerase chain reaction (RT-PCR)

Surgical specimens were processed immediately after operation. Total RNAs were extracted from tissues by using Trizol reagent (Invitrogen, Carlsbad, USA) according to the manufacturer’s protocol. All procedures were performed with i-cyclor. 1.0 g of total RNA was transcripted with River-Tra Ace (Toyobo, Tokyo, Japan), Oligo (dT) 20, RNase inhibitor, 5-RT buffer, and dNTP mixture. RT-PCR was performed at 42°C for 20 min and then at 95°C for 5 min using 1.0 g of RNA per reaction. The cDNA was amplified with TaKaRa Taq (TaKaRa, Ootsu, Japan), according to the manufacturer’s protocol. The PCR products were separated by electrophoresis using 1.5% agarose gels (sample volume: 10 μL, voltage: 100 V) and visualized by ethidium bromide staining for 10 min and ultraviolet illumination (Kodak, New Haven, USA). The following specific primers were used: HMGB1, sense strand: 5’-TATGGCAAAAGCGGACAAGG-3’, antisense strand: 5’- CTTCGCAACATCACCAATGGA-3’; GAPDH, sense strand: 5’-ATCAGCAATGCCTCCTGCAC-3’, antisense strand: 5’-CGTCAAAGGTGGAGGAGTGG-3’. Human GAPDH served as an internal control for the efficiency of mRNA isolation and cDNA synthesis.

### Western blot analysis

The fresh tissues were clipped, washed three times with ice-cold phosphate-buffered saline (PBS), and then the samples were lysed on ice in RIPA buffer with protease inhibitors and quantified to the same amount (30 μg). The proteins were separated by 12% SDS–PAGE and transferred to a PVDF membrane (GE healthcare, USA). After being blocked with 5% milk for 1 hour at room temperature, the membranes were then incubated overnight at 4°C with anti-HMGB1 (1:500) (Abcam, Cambridge, MA, USA) or anti-GAPDH (1:1000) (Santa Cruz Biotechnology, USA) antibodies. Followed by anti-mouse or rabbit horseradish peroxidaseconjugated IgG, an ECL kit (GE healthcare, USA) was used for detection.

### Immunohistochemistry (IHC)

The expression of HMGB1 in tumor tissues of the cohort of 161 patients, as well as 60 available para-tumor tissues from the same cohort, was examined by Immunohistochemistry (IHC). The samples were fixed by formalin, embedded in paraffin and cut into 4-μm thick sections. Then, the sections were de-waxed in xylene and rehydrated with ethanol arranged a graded concentration. After blocked with 0.3% hydrogen peroxide, the antigens were retrieved in a microwave in 10 mM citrate buffer (pH 6.0) for 30 minutes and cooled to room temperature. After washing with PBS, the sections were incubated overnight at 4°C with mouse monoclonal antibody against human HMGB1 with a dilution of 1:300 (Abcam, Cambridge, MA, USA). Subsequently, horseradish peroxidase conjugated secondary antibody was used. The sections were developed with diaminobenzidine tetrahydrochloride (DAB) and counterstained with hematoxylin. As-known HMGB1 positive colorectal cancer specimens were selected as positive controls [24]. Negative controls were employed in which the primary antibody was replaced by PBS.

### IHC evaluation

To estimate the expression of HMGB1, five fields were selected and at least expression of 1,000 tumor cells in sum, the evaluation used a high-power microscopy. The expression of HMGB1 in HCC was scored with the proportion of positive cells and using intensity. We ranked the proportion of positive cells into 4 categories: 1 (≤25%), 2 (26%-50%), 3 (51%-74%), and 4 (≥75%). Then we evaluated the intensity of nuclear or cytoplasmic staining and grouped them into the following four categories: no staining/background of negative controls (score = 1), weak staining detectable above background (score = 2), moderate staining (score = 3), and intense staining (score =4). The index was obtained by multiplying the intensity and percentage scores, the results as below: (-), (+), (++), (+++), (++++) indicated multiply-indexes of 1-2, 3-4, 6-8, 9-12 and 16, respectively; (-), (+), (++) were defined as low expression, and (+++) and (++++) were defined as high expression. Each section was independently scored by two pathologists. If an inconsistency occurred, a third pathologist was consulted to achieve consensus. The IHC score methods were identical for tumor and para-tumor tissues. This evaluation method was modified from the method used by Peng et al [24].

### Follow up

Follow-up of patients included physical examination, routine laboratory testing, and contrast-enhanced abdominal computed tomography every 3 months in the first 2 years, and every 6 months in 3 to 5 years after surgery, then every year thereafter. At each follow-up visit, liver function tests and alpha fetoprotein (AFP) were determined. Chest radiography was done every 6 months to observe lung metastasis. If necessary, CT of the chest, bone scintigraphy and positron emission tomography (PET) were also performed for the diagnosis of metastasis and/or recurrence. The last follow-up date for patients still alive was November 2011.

Causes of death and sites of recurrence were determined from death certificates, medical interviews, and radiological findings. Overall survival was defined as the interval between the time of hepatectomy to death or to the last date of follow-up. Disease-free survival time was between the time of hepatectomy and the time when recurrence was diagnosed or to the time of the last follow-up. The treatment for recurrent tumor was determined by our multidisciplinary team (MDT) including surgeons, oncologists, radiologists, gastroenterologists, and pathologists.

### Statistical analysis

The statistical analyses were performed using the SPSS 13.0 statistical software (SPSS Company, Chicago, Illinois, USA). Comparisons between 2 groups were done using the student’s t-test for continuous data and the Chi square test for categorical data. The correlation between the HMGB1 expression and clinicopathologic characteristics were analyzed with the Chi square test. The overall and disease-free survivals were calculated by Kaplan-Meier method and compared by log-rank test. The prognostic varieties in predicting overall and disease-free survival were assessed by multivariate Cox proportional hazards regression analysis. Results were given as mean ± S.D. All statistical tests were two-sided, and a significant difference was considered when *p* < 0.05.

## Results

### Increased HMGB1 mRNA and protein expression in HCC tissue

**Figure 1:**
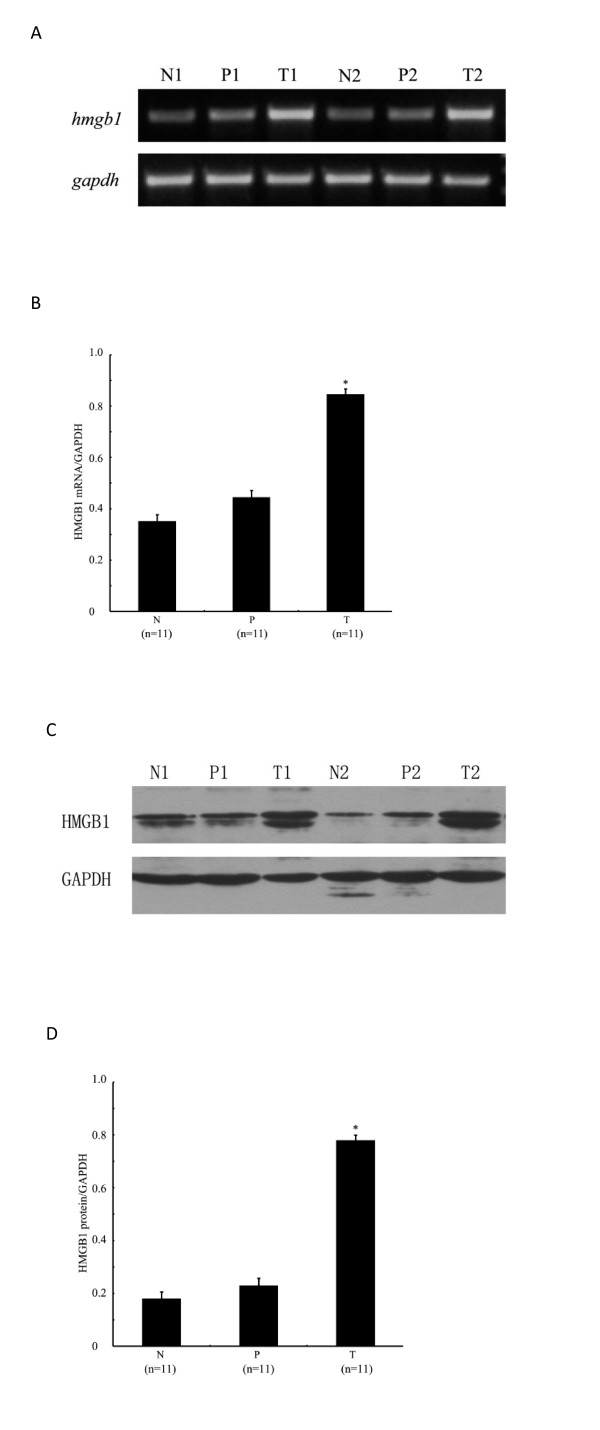
**RT-PCR and Western blot analysis of HMGB1 expression in normal (N), para-tumor (P) and tumor tissue (T) of all 11 HCC cases fresh tissues.** (**a**) Representative RT-PCR results of two cases, and (**c**) Representative Western blot results of two cases. (**b**) Relative quantity of HMGB1 mRNA expression, and (**d**) protein expression. Each value which represents the average of 11 cases expressed as the mean ± SE. Human GAPDH served as an internal control, the gray-scale comparison between HMGB1 and GAPDH was used to quantify the bands of RT-PCR and western blot results. * *p* < 0.001, compare to normal (N) and para-tumor (P).

The expression of HMGB1 mRNA and protein were detected and analyzed in all fresh tissues from 11 HCC patients. The RT-PCR results showed that HMGB1 mRNA level was significantly higher in tumor tissue, compared to that in para-tumor tissue (0.848 ± 0.075 Vs 0.446 ± 0.102, *p* < 0.001) and that in normal tissue (0.848 ± 0.075 Vs 0.354 ± 0.081, *p* < 0.001). The Western blot analysis of HMGB1 protein also showed that the expression of HMGB1 protein was significantly higher in tumor tissue, compared to that in para-tumor tissue (0.781 ± 0.105 Vs 0.230 ± 0.070, *p* < 0.001) and that in normal tissue (0.781 ± 0.105 Vs 0.180 ± 0.062, *p* < 0.001, Figure 1).

**Figure 2:**
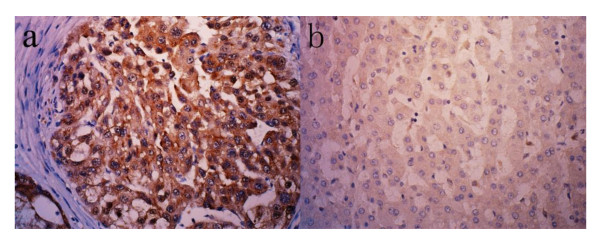
**Representative immunohistochemical staining of HMGB1 in tumor and para-tumor tissue.** (**a**) Over expression of HMGB1 in tumor tissue, HMGB1 immunostaining was predominantly located in cytoplasm of tumor cells (×400); (**b**) Low expression of HMGB1 in para-tumor tissue (×400).

Immunohistochemistry was performed in all 161 paraffin-embedded, archival HCC tumor samples and in available 60 para-tumor samples. Positive HMGB1 immunostaining was predominantly observed in the cytoplasm of carcinoma cell, and rarely in nucleus. High expression of HMGB1 was detected in 69/161(42.9%) of tumor tissues, and only 5/60(8.3%) in para-tumor tissues (Figure 2). Among tumor samples, HMGB1 expression was scored 1–2 in 13 (8.1%), 3–4 in 21 (13.0%), 6–8 in 58 (36.0%), 9–12 in 50 (31.1%) and 16 in 19 (11.8%) specimens.

### Correlation of HMGB1 protein expression with the clinicopathologic characteristics

The association between HMGB1 protein expression and clinicopathological characteristics of HCC was explored by the Chi square test. As it was showed in Table 1, high expression of HMGB1 was significantly associated with incomplete encapsulation (*p* = 0.035) and advanced TNM stage (*p* = 0.036). However, no significant relationship was found between HMGB1 protein expression and variables such as gender, age, HBV infection, alpha fetoprotein (AFP) level, underlying liver cirrhosis, Child–Pugh classification, tumor size, tumor number and tumor differentiation (Table 1).

### Correlation of HMGB1 protein expression with survivals

**Figure 3:**
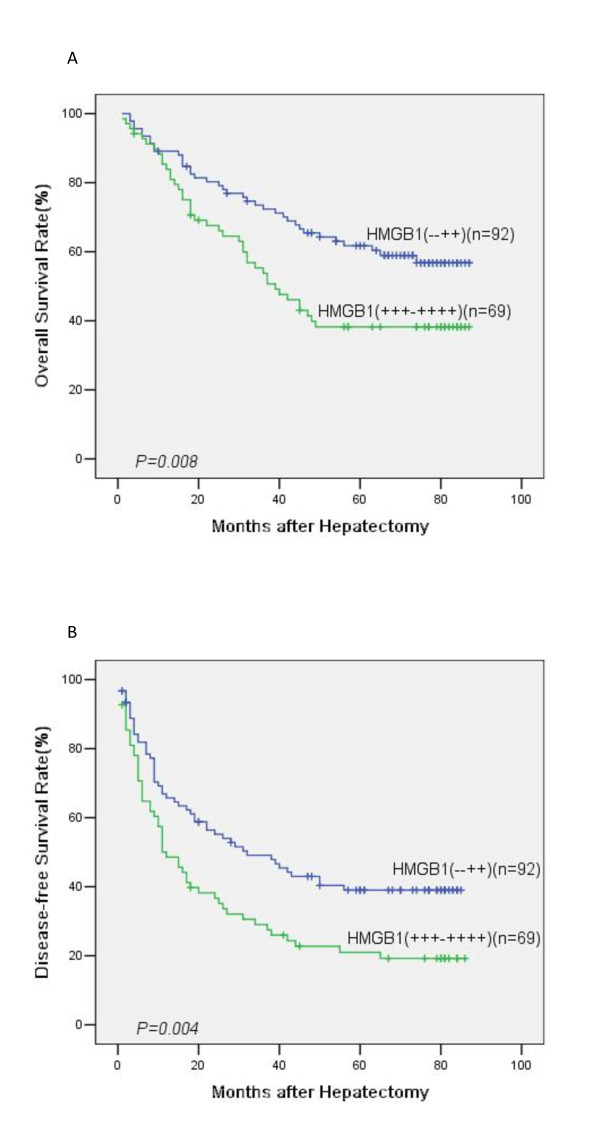
**Overall and disease-free survival curves of 161 HCC cases after curative hepatectomy assessed by Kaplan–Meier analysis according to HMGB1 expression.** Patients with high expression of HMGB1 were significantly associated with poorer overall survival (**a**, *p* = 0.008) and disease-free survival (**b**, *p* = 0.004).

The mean follow-up period was 47.5 ± 17.9 months (range 6.0-87.0 months). At the end of follow up, there were 78 deaths and 83 survivals. The 1, 3, 5-year overall survival of the whole group was 86.9%, 64.4% and 51.7% respectively. Univariate analysis showed that the overall survival was directly influenced by tumor size (*p* = 0.005), tumor number (*p* = 0.003), TNM stage (*p* = 0.004), and expression of HMGB1 (*p* = 0.008). Factors not significantly affecting overall survival included age, gender, HBV infection, AFP level, underlying liver cirrhosis, Child-pugh classification, tumor encapsulation and tumor differentiation. Multivariate analysis showed that expression of HMGB1 (*p* = 0.009, HR = 1.834; 95%CI: 1.167-2.881) and tumor size (*p* = 0.005, HR = 1.902; 95%CI: 1.209-2.992) were independent prognostic factors for overall survival (Table 2). The 1, 3, 5-year overall survival for patients with HMGB1 high expression was 83.9%, 53.8%, 38.2% respectively, and 89.1%, 72.4%, 61.7% respectively for patients with HMGB1 low expression (*p* = 0.008, Figure 3).

**Table 2:** Univariate and Multivariate analyses of overall and disease-free survival for 161 HCC patients

**Variables**	**Univariate analysis**		**Multivariate analysis**	
	**Chi-Square (χ**^**2**^)	***P *value**	**HR (95% CI)**	***P *value**
**Overall survival**				
Age(≤55y vs >55y)	0.453	0.501		
Gender(male vs female)	1.875	0.171		
HBV infection(absent vs present)	0.011	0.915		
AFP level(≤400 ng/ml vs >400 ng/ml)	0.170	0.680		
Liver cirrhosis(absent vs present)	1.631	0.202		
Child-pugh(A vs B)	0.158	0.691		
Tumor size(≤5 cm vs >5 cm)	7.962	**0.005**	1.902(1.209-2.992)	**0.005**
Tumor number(single vs multipule)	8.763	**0.003**	0.601(0.240-1.504)	0.277
Tumor encapsulation(complete vs incomplete)	2.590	0.108		
TNM stage(I-II vs III)	8.484	**0.004**	1.567(0.771-3.184)	0.215
Tumor differentiation(I-II vs III-IV)	0.033	0.856		
HMGB1(low vs high)	7.022	**0.008**	1.834(1.167-2.881)	**0.009**
**Disease-free survival**				
Age(≤55y vs >55y)	0.812	0.367		
Gender(male vs female)	0.120	0.729		
HBV infection (absent vs present)	0.118	0.731		
AFP level(≤400 ng/ml vs >400 ng/ml)	0.748	0.387		
Liver cirrhosis(absent vs present)	1.972	0.160		
Child-pugh(A vs B)	1.502	0.220		
Tumor size(≤5 cm vs >5 cm)	8.367	**0.004**	1.661(1.119-2.465)	**0.012**
Tumor number(single vs multipule)	14.484	**<0.001**	0.484(0.218-1.071)	0.073
Tumor encapsulation(complete vs incomplete)	6.531	**0.011**	0.739(0.497-1.099)	0.136
TNM stage(I-II vs III)	11.494	**0.001**	1.314(0.719-2.402)	0.374
Tumor differentiation(I-II vs III-IV)	3.062	**0.080**	1.252(1.088-2.419)	0.291
HMGB1(low vs high)	8.176	**0.004**	1.622(1.088-2.419)	**0.018**

HBV: hepatitis B virus, AFP: alpha fetoprotein, HMGB1: high mobility group box 1, HR: Hazard Ratio.

The 1, 3, 5-year disease-free survival of the whole group was 58.2%, 40.3% and 31.3% respectively. Univariate analysis showed that tumor size (*p* = 0.004), tumor number (*p* < 0.001), tumor encapsulation (*p* = 0.011), TNM stage (*p* = 0.001) and expression of HMGB1 (*p* = 0.004) were prognostic factors for disease-free survival. Multivariate analysis indicated that expression of HMGB1 (*p* = 0.018, HR = 1.622; 95%CI: 1.088-2.419) and tumor size (*p* = 0.012, HR = 1.661; 95%CI: 1.119-2.465) were independent prognostic factors for disease-free survival (Table 2). The 1, 3, 5-year disease-free survival for patients with HMGB1 high expression was 48.6%, 29.0%, 21.0% respectively, and 65.7%, 49.1%, 39.9% respectively for patients with HMGB1 low expression (*p* = 0.004, Figure 3).

**Figure 4:**
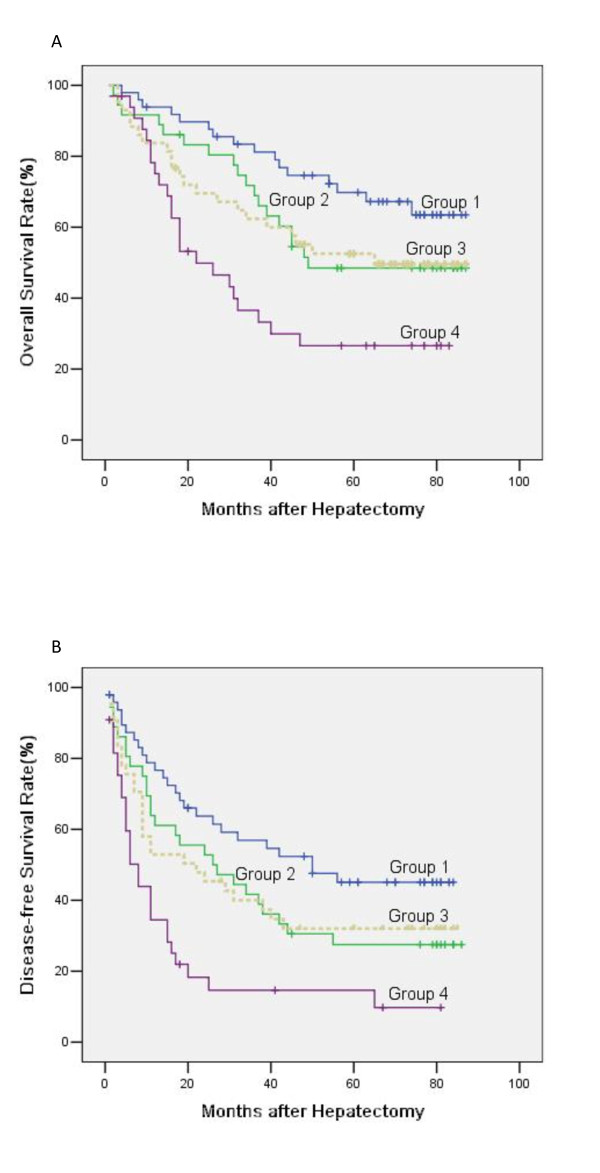
**Overall and disease-free survival curves assessed by Kaplan–Meier analysis according to HMGB1 expression and tumor size.** (**a**) Overall survival. High expression of HMGB1 predicted poorer overall survival only for tumor >5 cm (group 3 VS group 4; *p* = 0.031); but not for tumor ≤5 cm (group 1 VS group 2; *p* = 0.101). (**b**) Disease-free survival. High expression of HMGB1 predict poor disease-free survival only for tumor >5 cm (group 3 VS group 4; p = 0.012); but not for tumor ≤5 cm (group 1 VS group 2; p = 0.087). Group 1, tumor ≤5 cm/ HMGB1 low expression (n = 49); group 2, tumor ≤5 cm/ HMGB1 high expression (n = 36); group 3, tumor >5 cm/ HMGB1 low expression (n = 43); group 4, tumor >5 cm/ HMGB1 high expression (n = 33).

In subgroup analysis, for 76 patients with tumor >5 cm, the 1, 3, 5-year overall survival for patients with HMGB1 high expression was 75.1%, 36.6%, 26.6% respectively, and 74.3%, 62.4%, 52.5% respectively for patients with HMGB1 low expression (*p* = 0.031), the corresponding disease-free survival was 34.5%, 14.6%, 14.6% respectively, and 52.9%, 40.0%, 32.0% respectively (*p* = 0.012, Figure 4). However, for patients with tumor ≤5 cm, the 1, 3, 5-year overall survival for patients with HMGB1 high expression was 91.7%, 68.9%, 48.5% respectively, and 91.8%, 81.2%, 69.8% respectively for patients with HMGB1 low expression (*p* = 0.101), and the corresponding disease-free survival was 61.1%, 41.7%, 27.5% respectively, and 76.7%, 56.9%, 45.1% respectively (*p* = 0.087, Figure 4).

## Discussion

In present study, we demonstrated that the expression of HMGB1 was significantly higher in HCC tissue, compared to that in para-tumor and normal liver tissue, high expression of HMGB1 was significantly associated with incomplete tumor encapsulation and advanced TNM stage, and for the first time, we revealed that high expression of HMGB1 predicted poorer survival for patients with HCC after curative hepatectomy, especially for patients with tumor >5 cm. Our results were in agreement with previous studies of other malignancies, including gastric cancer, colorectal cancer, prostate cancer and nasopharyngeal carcinoma et al, in which overexpression of HMGB1 in tumor tissue has been observed and a correlation between overexpression of HMGB1 and poorer prognosis has been established [18-21].

There was increasing interest in the role of HMGB1 in HCC in recent years. Cheng et al [25] firstly reported the correlation between serum HMGB1 level and clinicopathologic features in patients with HCC, higher serum HMGB1 level correlated with bigger tumor size, poor tumor differentiation and advanced TNM stage. Then Jiang et al [22] reported that overexpression of HMGB1 in tumor tissue, rather than in para-tumor and normal tissue, correlate with advanced TNM stage, vascular invasion and capsule invasion by detecting fresh samples from 34 HCC patients. Similarly, our study, with immunohistochemistry in a cohort of consecutive 161 previously untreated HCC, demonstrated that high expression of HMGB1 was significantly associated with incomplete tumor encapsulation and advanced TNM stage. Since only the patients without vascular invasion were enrolled, the correlation between the expression of HMGB1 and vascular invasion cannot be analyzed in present study.

Different models indicated the HMGB1 protein had beneficial influence on tumor development. HMGB1 protein was constitutively expressed in the nucleus of tumor cells, and also can be released by inflammatory cells and by tumor cells [26]. Constant release of HMGB1 as a proinflammatory cytokine from necrotic tumor cells would create a microenvironment similar to chronic inflammations, and this condition was known to contribute to the development of epithelial malignancies [27]. In present study, we conducted the RT-PCR, Western blot and immunohistochemical methods to detect the expression of HMGB1 in tumor, para-tumor and normal tissues. Our results showed that the expression of HMGB1 was significantly higher in tumor tissue than that in para-tumor and normal tissue, which indicated that HMGB1 might play an important part in the carcinogenesis of HCC.

HMGB1 can function during inflammation, tumor invasion and metastasis through its receptors: RAGE and TLRs. Liang et al [28] reported that knockdown of RAGE inhibited expression of VEGF and SP1 protein in colorectal cancer cells, and silence of RAGE expression effectively inhibited colorectal cancer angiogenesis. HMGB1 also takes part in immune system through TLRs. TLRs exist in almost immunosuppressive cells and recent study showed that tumor cell-derived HMGB1 might suppress naturally acquired CD8 T cell-dependent antitumor immunity via enhancing Treg to produce IL-10, which is necessary for Treg-mediated immune suppression [29]. These results indicated that HMGB1 played an active role in tumor immune suppression, which promoted the development of tumor invasion and metastasis. In present study, we demonstrated that high expression of HMGB1 was associated with incomplete tumor encapsulation and advanced TNM stage. Studies on other cancers also showed that HMGB1 expression was positively correlated with lymph node metastasis and distant metastasis. It indicated that overexpression of HMGB1 was associated with tumor growth and invasion [22].

Hepatectomy is the most effective curative therapy and provides better survival outcomes for patients with HCC. Unfortunately, approximately 33% of HCC patients die within the first year even after curative surgery, mainly because of tumor recurrence and spread [2,3]. Currently, prognostic evaluation is mainly based on tumor stage and histopathologic observation such as tumor size, tumor number, and vascular invasion [1-4]. However, we found that although patients have modest tumor presentation, the prediction for patients’ overall and disease-free survival can be variable and inaccurate. Recent studies have suggested some factors, such as molecular and cellular characteristics of primary tumor, may improve our ability to prognosticate [4]. In present study, the expression of HMGB1 was revealed as an independent prognostic factor for both overall and disease-free survival for patients with HCC after curative hepatectomy. The patients with high expression of HMGB1 had a shorter overall and disease-free survival. More importantly, subgroup analysis showed the expression of HMGB1 was significantly associated with poor prognosis in patients with HCC >5 cm, but not in patients with HCC ≤5 cm. Studies in other cancers, including colorectal cancer, nasopharyngeal carcinoma, and squamous-cell carcinoma of the head and neck, also showed that HMGB1 expression was inversely correlated with survival in late stage cancers but not in early stage caners [18-21]. This trend suggested that HMGB1 might be an important prognostic marker for late stage HCC after hepatectomy. However, prospective clinical studies are needed to confirm that HMGB1 is one of the reliable clinical predictors of outcome for individual patients with HCC undergoing hepatectomy.

## Conclusions

In conclusion, our study revealed that HMGB1 is an independent prognostic factor for overall and disease-free survival in patients with HCC after curative hepatectomy. High expression of HMGB1 in tumor is strongly correlated with incomplete tumor encapsulation and advanced TNM stage. However, these results, which are based on a Chinese cohort (all surgical patients without vascular invasion and mostly associated with HBV infections), should be further confirmed in other populations of patients with HCC. Our findings suggest that HMGB1 might be used as a new biomarker and a potential therapeutic target for HCC.

## Abbreviations

HMGB1, high mobility group box 1; HCC, hepatocellular carcinoma; RAGE, receptor for advanced glycation end-products; TLRs, Toll like receptors; AFP, alpha-fetoprotein; HBV, hepatitis B virus; RT-PCR, Reverse transcription-polymerase chain reaction; PBS, phosphate-buffered saline; TNM, tumor-node-metastasis; IHC, immunohistochemistry; HR, Hazard Ratio.

## Competing interests

The authors declare that they have no competing interests.

## Authors’ contributions

FRL, YJZ and MSC are responsible for study design, experiments, data analysis and interpretation, and draft the manuscript. ZWP, HJG, and LX are participated in study design, data analysis and interpretation. All authors read and approved the final manuscript.
